# The Long Tail of Long COVID-19: Broad and Extensive Increase in Utilization Within an Integrated Healthcare System

**DOI:** 10.7759/cureus.106676

**Published:** 2026-04-08

**Authors:** Dustin W Ballard, E. Margaret Warton, Jacek Skarbinski, Marcos H Siqueiros, S. Madhavi Cholleti, David R Vinson, Dustin G Mark, Edward J Durant, Daniel D DiLena, Mary E Reed

**Affiliations:** 1 Bernard J. Tyson School of Medicine, Kaiser Permanente, Pasadena, USA; 2 Emergency Medicine, The Permanente Medical Group, Pleasanton, USA; 3 Division of Research, Kaiser Permanente Northern California, Pleasanton, USA; 4 Infectious Diseases, The Permanente Medical Group, Pleasanton, USA; 5 Internal Medicine, The Permanente Medical Group, Pleasanton, USA

**Keywords:** covid-19, healthcare utilization, integrated healthcare system, long covid, pasc

## Abstract

Background: The impact of the emergence of Long COVID on healthcare utilization in a U.S. community setting remains underexplored.

Objective: To examine the healthcare utilization of Long COVID patients compared with pre-COVID-19 era controls before and after SARS-CoV-2 infection within a large integrated delivery system.

Methods: This was a retrospective cohort study of adult patients with documented SARS-CoV-2 infection (1/1/2021-6/30/2022) in a multi-site Northern California health system. Long COVID diagnosis was defined by at least one encounter with an associated International Classification of Diseases, Tenth Revision code and/or referral to Long COVID specialty care. Utilization outcomes included inpatient/outpatient encounters, laboratory/imaging/functional testing, specialty care referrals/encounters, and medication use. We used propensity-weighted difference-in-differences models adjusted for study month to compare outcomes between baseline and post-SARS-CoV-2 infection (Long COVID window).

Results: Among 600,295 patients with 635,230 SARS-CoV-2 episodes, 3,545 met criteria for Long COVID care-seeking. Long COVID patients had higher baseline utilization and greater increases in post-diagnosis utilization in adjusted analyses. After propensity weighting, these differences persisted and were consistent across the study period, particularly in cardiac and pulmonary care. Total encounters per year were 29.9 post and 14.1 pre (Long COVID) versus 17.7 post and 12.5 pre (NO Long COVID). Total annual ED visits per 100 patients were 65.1 post and 38.0 pre (Long COVID) versus 38.0 post and 32.0 pre (NO Long COVID). Annual hospitalizations per 100 patients were 10.5 post and 5.3 pre (Long COVID) versus 7.4 post and 5.5 pre (NO Long COVID).

Conclusion: Long COVID patients had higher healthcare utilization before and after diagnosis, with sharper increases across nearly all outcomes.

## Introduction

Long COVID, also known as post-acute sequelae of SARS-CoV-2 infection (PASC), is a condition where symptoms persist for at least four to 12 weeks after an initial SARS-CoV-2 infection. While the majority of patients with Long COVID eventually recover, many require longitudinal, multidisciplinary care to address a wide range of symptoms affecting multiple organ systems [1]. Common symptoms can include chronic or prolonged post-viral fatigue, cognitive dysfunction ("brain fog"), shortness of breath, autonomic dysfunction, and psychological distress. Evidence suggests that healthcare utilization for Long COVID patients is higher following their diagnosis and when compared with the general population [2-5]. Estimates of this utilization burden vary across study settings and designs, and its impact appears to be broad and involves multiple specialties, including emergency medicine, adult primary care, pulmonology, cardiology, neurology, rheumatology, and mental health services. Studies from the UK estimate the utilization and cost-impact of Long COVID in their system to be significantly higher than for those not afflicted, and studies have reported increased healthcare utilization across multiple care settings, including primary care, specialty clinics, emergency departments (EDs), and inpatient settings [6,7].

Long COVID necessitates a coordinated, interdisciplinary approach to care. However, an evidence gap exists with regards to how best to design and implement such an approach in a way that effectively manages the health of patients with Long COVID while also mitigating the health system burden they may pose. The primary objective of this study was to assess differences in healthcare utilization before and after SARS-CoV-2 infection among patients who subsequently sought care for Long COVID compared with those who did not, within a large US integrated health delivery system. We examined the outcomes of patients who sought clinical care for Long COVID symptoms across multiple utilization domains, including inpatient and outpatient encounters, emergency department visits, specialty care referrals, diagnostic testing, medication use, and work-impact documentation, using propensity-weighted difference-in-differences models to compare pre-pandemic baseline utilization (2019) with the 12-month period following 28 days after a COVID diagnosis.

## Materials and methods

Setting

This was a retrospective cohort study of adult patients with SARS-CoV-2 infection (COVID-19) between January 1, 2021, and June 30, 2022, in a community-based comprehensive healthcare system delivering care to 4.4 million members at 21 hospitals with associated medical clinics (Kaiser Permanente Northern California (KPNC)). Members are representative of the ethnic and socioeconomic diversity of the surrounding population [8]. The KPNC system is supported by a comprehensive, integrated electronic health record (EHR) that includes inpatient, outpatient, emergency, pharmacy, laboratory, and imaging data as well as claims data [9]. KPNC is a learning healthcare system with an applied research agenda [10]. The KPNC Institutional Review Board approved the study and waived informed consent. During the study period, SARS-CoV-2 testing was performed broadly in the system on an outpatient and inpatient basis, and when members self-reported positive results of at-home testing, these results were documented in the EHR.

Study population

The study cohort included all COVID episodes among health plan members 18 years or older identified through a COVID-19 diagnosis and/or recorded SARS-CoV-2 infection (as identified via EHRs). For each COVID episode, an index month was defined as the date of COVID diagnosis for non-hospitalized patients and the date of discharge for hospitalized patients. Patients who died or had fewer than three months of active membership prior to the index month were excluded. In our system, nirmatrelvir/ritonavir became available and distributed to eligible patients starting in December 2021.

Exposure

Each COVID episode was assigned to the Long COVID cohort if it was followed by a qualifying Long COVID healthcare seeking utilization event, defined as either: 1) referral to Long COVID clinic; 2) attendance at a Long COVID class; or 3) a new diagnosis or problem list entry of Long COVID (International Classification of Diseases, Tenth Revision, Clinical Modification U09.9). To qualify as Long COVID care-seeking (distinct from care provided for the index COVID infection), we required that the Long COVID diagnosis/care-seeking occur at least 28 days after a documented COVID diagnosis and/or positive SARS-CoV-2 test. Referrals to a Long COVID consult service were made by primary care clinicians and could include a Long COVID clinic or class at the referring provider’s discretion. We chose the 28-day cut-off as that was the temporal definition used by Long COVID providers in our system during the study period. A health plan member could be included in both study groups depending on how many qualifying COVID episodes they had and the timing of their Long COVID diagnosis.

Utilization outcomes

We collected utilization outcomes from the EHR, defined as any healthcare encounter in the 12-month Long COVID outcome period, including outpatient (primary care or specialty care) referrals and encounters, ED visits, hospitalizations, laboratory testing, imaging or functional testing, and pharmacy prescriptions. We also collected the occurrences of discrete patient-requested physician release-from-work notes that were created and filed in the EHR.

Analysis

**Figure 1:**
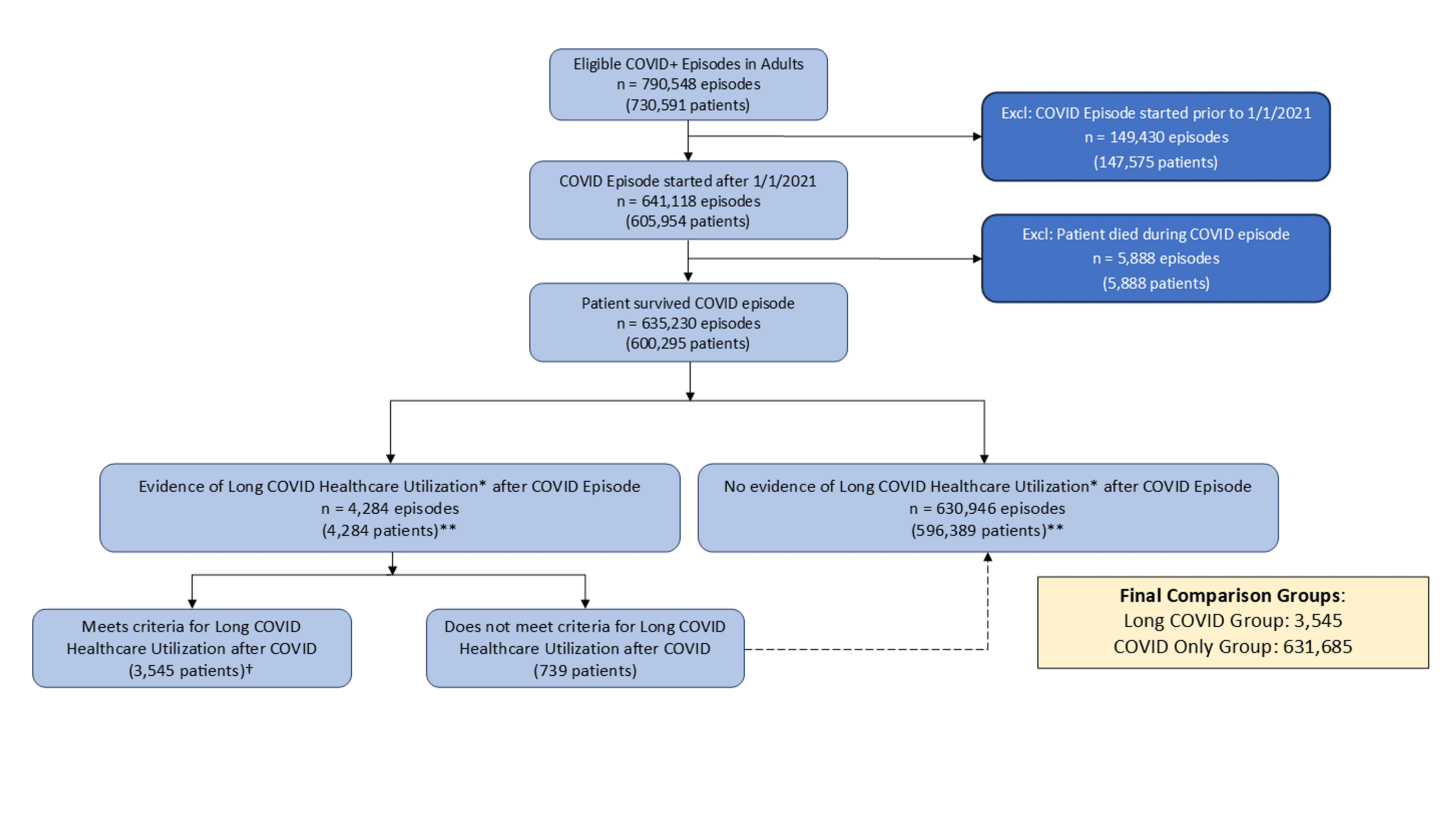
Cohort Assembly Diagram: Long COVID Healthcare Utilization Episodes January 1, 2021, Through June 30, 2022. *Long COVID Healthcare Utilization identified as any referral to Long COVID clinic, attendance at Long COVID class, diagnosis entered, problem list entry created. **Patients can be counted in both Long COVID and COVID Only (No Long COVID) groups depending on timing of multiple COVID episodes and Long COVID Healthcare Utilization. †Patients cannot have multiple Long COVID episodes under our definition.

We compared healthcare utilization outcomes during 2019 as a baseline period (prior to the COVID-19 pandemic) and the year following COVID infection+28 days (Long COVID window) between episodes with Long COVID (cohort) and episodes with NO Long COVID (COVID Only). The Cohort Assembly is shown in Figure 1. To create unbiased comparison groups, we used a propensity score model with inverse probability weights and difference-in-differences (DID) models that adjusted for study month. Such DID models assess the effect of a condition or intervention (here a Long COVID diagnosis) applied to another group (NO Long COVID) by comparing their outcomes in terms of two differences. Our DID analyses estimate the causal effect of the Long COVID condition by using propensity weighting to balance potential confounders (including baseline comorbidity burden) between the two study groups and adjusting for temporal trends during the study period [11]. Predictors of Long COVID in our system had been previously identified in our cohort of patients and are shown in Table 1 and Table 2 [12]. Propensity matching variables were chosen from these if they were reliably available at the start of our cohort assembly timeframe (2019). These included age, sex, race-ethnicity, insurance type, the standardized 2019 Neighborhood Deprivation Index (NDI), smoking status, body mass index, Elixhauser comorbidity score, count of active problem list diagnoses, and count of active medications from the medication list. We used General estimating equation models to account for the longitudinal nature of the data with normal distributions and compound symmetry covariance structure. Statistical analyses were performed with SAS software, Version 9.4 (SAS Institute Inc., Cary, NC, USA).

**Table 1:** Demographic Characteristics of Cohort *Variables marked with an asterisk were included in propensity score matching. Abbreviations: SD = standard deviation, IQR = interquartile range.

Demographic Characteristic	Overall	Long COVID Care* (%)	No Long COVID Care (%)	P-Value	Effect Size
N	(N=635,230)	(N=3545)	(N=631,685)		
Age, years*					
Mean (SD)	46.3 (16.5)	50.9 (15.0)	46.3 (16.5)	<0.001	0.29
Median (interquartile range)	44.5 (33.3-58.1)	50.4 (39.9-61.3)	44.4 (33.3-58.1)	----	----
Range	18.0-106.3	18.0-100.3	18.0-106.3	----	----
Age, category*				<0.001	0.02
18-29	112,223 (17.7)	281 (7.9)	111,942 (17.7)		
30-39	143,686 (22.6)	617 (17.4)	143,069 (22.6)		
40-49	129,542 (20.4)	843 (23.8)	128,699 (20.4)		
50-59	110,661 (17.4)	811 (22.9)	109,850 (17.4)		
60-75	105,311 (16.6)	765 (21.6)	104,546 (16.6)		
75 and older	33,807 (5.3)	228 (6.4)	33,579 (5.3)		
Race/Ethnicity*				<0.001	0.02
Black	48,214 (7.6)	320 (9.0)	47,894 (7.6)		
NH White	254,442 (40.1)	1671 (47.1)	252,771 (40.0)		
Asian	121,240 (19.1)	429 (12.1)	120,811 (19.1)		
Hispanic	161,948 (25.5)	896 (25.3)	161,052 (25.5)		
Other/Unknown	49,386 (7.8)	229 (6.5)	49,157 (7.8)		
Female*	364,870 (57.4)	2340 (66.0)	362,530 (57.4)	<0.001	0.01
Standardized 2019 Neighborhood Deprivation Index*					
Mean (SD)	-0.2 (0.9)	-0.1 (0.9)	-0.2 (0.9)	<0.001	0.07
Median (interquartile range)	-0.3 (-0.8-0.3)	-0.3 (-0.7-0.4)	-0.3 (-0.8-0.3)	<0.001	----
Range	-2.4-4.2	-1.9-4.1	-2.4-4.2	----	----
Missing, n (%)	365 (0.1)	3 (0.1)	362 (0.1)	----	----
Insurance coverage at COVID episode				<0.001	0.01
Medicare	43,575 (6.9)	302 (8.5)	43,273 (6.9)		
Medicaid	80,218 (12.6)	545 (15.4)	79,673 (12.6)		
Other	511,437 (80.5)	2698 (76.1)	508,739 (80.5)		

**Table 2:** Clinical Characteristics of Cohort *Variables marked with an asterisk were included in propensity score matching. Abbreviations: BMI = body mass index, PHQ-9 = Patient Health Questionnaire-9, ED = emergency department, IP = inpatient, ICU = intensive care unit, RA = room air, NC = nasal cannula, HFNC = high-flow nasal cannula, NIV = non-invasive ventilation, IV = invasive ventilation.

Clinical Characteristic	Overall (%)	Long COVID Care* (%)	No Long COVID Care (%)	P-Value	Effect Size
N	(N=635,230)	(N=3545)	(N=631,685)		
BMI Category*				<0.001	0.01
<30	306,937 (48.3)	1527 (43.1)	305,410 (48.3)		
30 and higher	221,092 (34.8)	1562 (44.1)	219,530 (34.8)		
Missing	107,201 (16.9)	456 (12.9)	106,745 (16.9)		
Smoking Status*				<0.001	0.01
Never	453,782 (71.4)	2512 (70.9)	451,270 (71.4)		
Current	33,760 (5.3)	137 (3.9)	33,623 (5.3)		
Former	127,909 (20.1)	839 (23.7)	127,070 (20.1)		
Passive/Unknown	19,779 (3.1)	57 (1.6)	19,722 (3.1)		
Diabetes at COVID episode start date	73,151 (11.5)	519 (14.6)	72,632 (11.5)	<0.001	0.01
Patient Health Questionnaire (PHQ-9) Scores					
PHQ-9 score in year before COVID					
Mean (SD)	7.7 (6.3)	9.1 (6.5)	7.7 (6.3)	<0.001	0.23
Median (interquartile range)	6.0 (2.0-12.0)	8.0 (4.0-14.0)	6.0 (2.0-12.0)	----	----
Range	0.0-27.0	0.0-27.0	0.0-27.0	----	----
PHQ-9 category year before COVID				<0.001	0.02
None/Minimal/Mild	68,332 (10.8)	478 (13.5)	67,854 (10.7)		
Mod/Mod Severe	29,474 (4.6)	286 (8.1)	29,188 (4.6)		
Severe	5700 (0.9)	68 (1.9)	5632 (0.9)		
No Survey	531,724 (83.7)	2713 (76.5)	529,011 (83.7)		
Categorized active diagnosis on problem list before COVID*				<0.001	0.03
None	45,801 (7.2)	99 (2.8)	45,702 (7.2)		
1-2	97,562 (15.4)	260 (7.3)	97,302 (15.4)		
3-6	194,010 (30.5)	872 (24.6)	193,138 (30.6)		
7-11	151,890 (23.9)	913 (25.8)	150,977 (23.9)		
12 or more	145,967 (23.0)	1401 (39.5)	144,566 (22.9)		
Disease Severity					
Highest level of COVID care				<0.001	0.08
None	219,522 (34.6)	557 (15.7)	218,965 (34.7)		
Virtual	300,947 (47.4)	1408 (39.7)	299,539 (47.4)		
Ambulatory	54,174 (8.5)	362 (10.2)	53,812 (8.5)		
ED	41,802 (6.6)	549 (15.5)	41,253 (6.5)		
IP	14,364 (2.3)	490 (13.8)	13,874 (2.2)		
IP with ICU	4421 (0.7)	179 (5.0)	4242 (0.7)		
Highest level of ventilation during COVID episode				<0.001	0.09
None	583,355 (91.8)	2416 (68.2)	580,939 (92.0)		
RA	37,030 (5.8)	500 (14.1)	36,530 (5.8)		
NC	8375 (1.3)	261 (7.4)	8114 (1.3)		
Mask	1664 (0.3)	45 (1.3)	1619 (0.3)		
HFNC	2201 (0.3)	162 (4.6)	2039 (0.3)		
NIV	1828 (0.3)	98 (2.8)	1730 (0.3)		
IV	777 (0.1)	63 (1.8)	714 (0.1)		
Therapeutics					
Vaccination Status before COVID				<0.001	0.03
None	197,634 (31.1)	1660 (46.8)	195,974 (31.0)		
Primary no booster	213,415 (33.6)	927 (26.1)	212,488 (33.6)		
Primary booster	199,824 (31.5)	815 (23.0)	199,009 (31.5)		
Incomplete	24,357 (3.8)	143 (4.0)	24,214 (3.8)		
On Metformin at COVID episode start	37,491 (5.9)	238 (6.7)	37,253 (5.9)	<0.05	0.00
Prevalent variant at COVID episode start				<0.001	0.03
Pre-Delta	87,300 (13.7)	831 (23.4)	86,469 (13.7)		
Delta	115,439 (18.2)	1023 (28.9)	114,416 (18.1)		
Omicron	432,491 (68.1)	1691 (47.7)	430,800 (68.2)		

## Results

**Figure 2:**
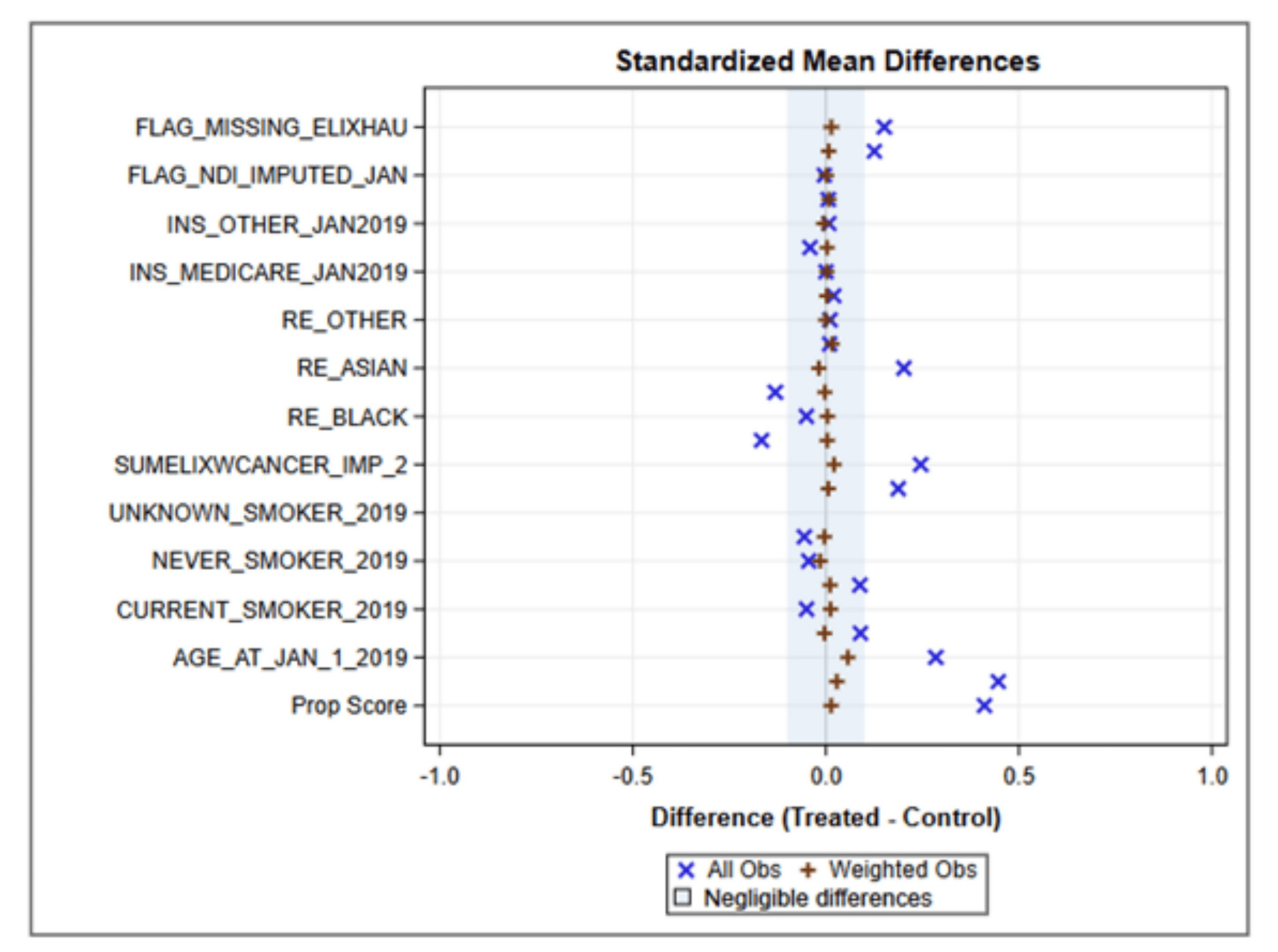
Propensity Score Standardized Mean Differences Plot.

**Figure 3:**
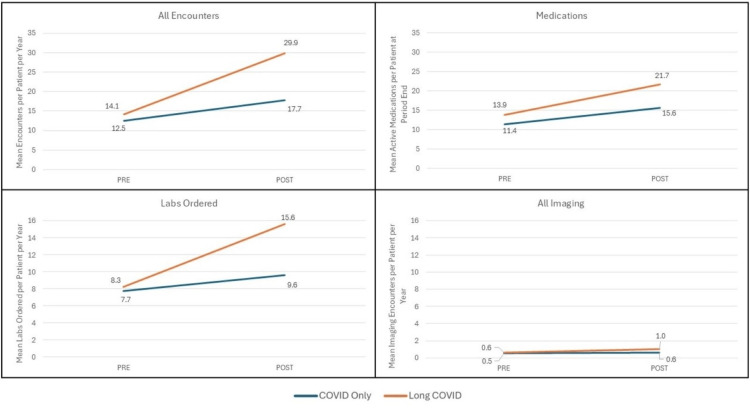
Pre- and Post-Comparisons Between the COVID-Only (NO Long COVID) and Long COVID Groups. We compared outcomes during 2019 and the year following COVID infection+28 days (Long COVID window) between those with Long COVID and those with COVID only using inverse probability of treatment weighting (IPTW) propensity score weights in models additionally adjusted for study month, time period, and exposure-time interaction terms to generate difference-in-differences model estimates. P<0.05 for all shown comparisons. Medication counts were defined as the number of medications that were marked as being "taken" on the medication list review at the encounter closest to 12/31/2019 for the pre period and the encounter closest to the end of the Long COVID window for the post period.

Our study cohort assembly is shown in Figure 1. The final comparison groups included 3545 unique patients (0.56%) in the Long COVID utilization group and 631,685 COVID episodes in the NO Long COVID group. Unadjusted analyses revealed broad differences across the comparison groups in changes in healthcare utilization between the pre and post periods, with the Long COVID group exhibiting higher utilization both before and after COVID. The propensity score standardized mean differences plot is shown in Figure 2. All of the weighted observations fall well within 0.01 standard deviations. After weighting adjustment, nearly all of the differences between the baseline and post-COVID period persisted. Figure 3 shows adjusted changes in healthcare utilization across four domains: Total health system encounters, completed laboratory and imaging orders, and active medications. 

**Figure 4:**
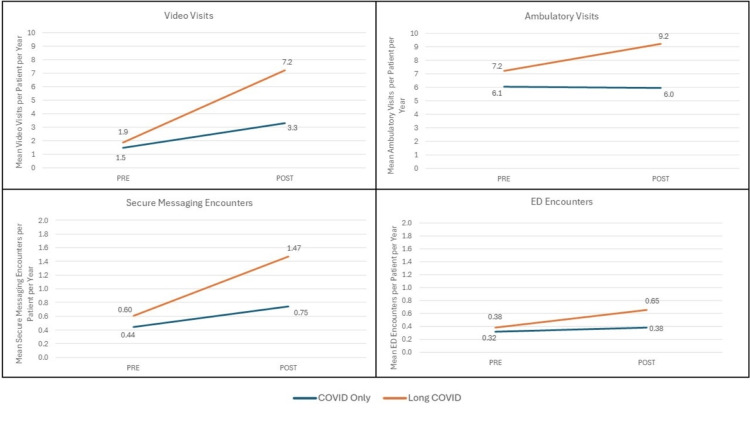
Pre- and Post-Comparisons Between the COVID-Only (NO Long COVID) and Long COVID Groups by Encounter Type. We compared outcomes during 2019 and the year following COVID infection+28 days (Long COVID window) between those with Long COVID and those with COVID only using inverse probability of treatment weighting (IPTW) propensity score weights in models additionally adjusted for study month, time period, and exposure-time interaction terms to generate difference-in-differences model estimates. P<0.05 for all shown comparisons.

Analysis of specific encounter types revealed pre-post COVID differences between Long COVID and non-Long COVID episodes across multiple types, including secure messaging, video visits, and ambulatory visits, as shown in Figure 4. Hospitalization events per 100 patients are not shown in the figure but were also more likely in the Long COVID group (Long COVID 10.5 [95% CI: 8.8,12.2] post/5.3 [95% CI: 4.3,6.3] pre versus NO Long COVID 7.4 [95% CI: 7.3,7.5] post/5.5 [95% CI: 5.4,5.5] pre). There was a non-significant difference in hospitalizations between the groups in the pre period.

**Figure 5:**
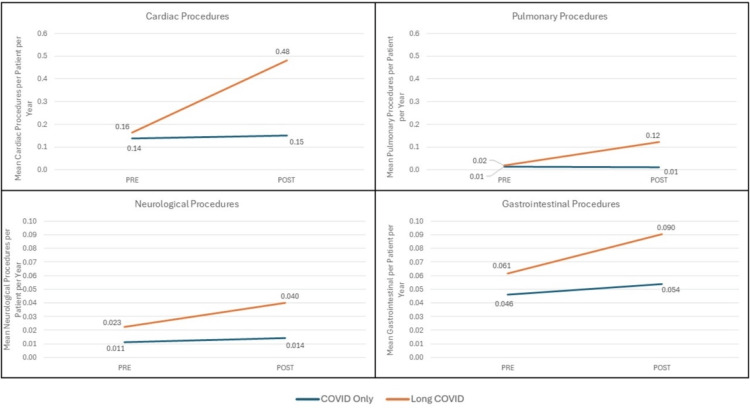
Pre- and Post-Comparisons Between the COVID-Only (NO Long COVID) and Long COVID Groups by Specialty Procedure Type. We compared outcomes during 2019 and the year following COVID infection+28 days (Long COVID window) between those with Long COVID and those with COVID only using inverse probability of treatment weighting (IPTW) propensity score weights in models additionally adjusted for study month, time period, and exposure-time interaction terms to generate difference-in-differences  model estimates. P<0.05 for all shown comparisons.

Increases in clinical domain specialty procedures were also more common among COVID episodes resulting in Long COVID. For example, the post-COVID unadjusted rate of obtaining transthoracic echocardiography (TTE) in the year following Long COVID episodes was nearly four times higher than in the year following COVID-only episodes despite having similar baseline rates in the pre-COVID period (Table 3). Adjusted results across all such specialty procedures, by organ system domain, are shown in Figure 5.

**Table 3:** Unadjusted Specialty Procedure Counts Abbreviations: SD = standard deviation.

Specialty Procedure	Overall	Long COVID	No Long COVID	P-Value	Effect Size
N	(N=635,230)	(N=3545)	(N=631,685)		
Pulmonary function test during base period, n (%)	8201 (1.3)	86 (2.4)	8115 (1.3)	<0.001	0.01
Pulmonary function test during base period, mean (SD)	0.03 (1.31)	0.08 (3.08)	0.03 (1.29)	0.26	0.02
Pulmonary function test during post period, n (%)	7331 (1.2)	421 (11.9)	6910 (1.1)	<0.001	0.08
Pulmonary function test during post period, mean (SD)	0.01 (0.17)	0.14 (0.40)	0.01 (0.17)	<0.001	0.40
Holter monitor during base period, n (%)	8345 (1.3)	93 (2.6)	8252 (1.3)	<0.001	0.01
Holter monitor during base period, mean (SD)	0.03 (0.24)	0.05 (0.33)	0.03 (0.24)	<0.001	0.08
Holter monitor during post period, n (%)	11,759 (1.9)	267 (7.5)	11,492 (1.8)	<0.001	0.03
Holter monitor during post period, mean (SD)	0.02 (0.19)	0.10 (0.39)	0.02 (0.19)	<0.001	0.25
Echocardiogram during base period, n (%)	12,207 (1.9)	103 (2.9)	12,104 (1.9)	<0.001	0.01
Echocardiogram during base period, mean (SD)	0.03 (0.22)	0.04 (0.25)	0.03 (0.22)	<0.01	0.05
Echocardiogram during post period, n (%)	18,099 (2.8)	402 (11.3)	17,697 (2.8)	<0.001	0.04
Echocardiogram during post period, mean (SD)	0.04 (0.26)	0.15 (0.46)	0.04 (0.25)	<0.001	0.29

**Figure 6:**
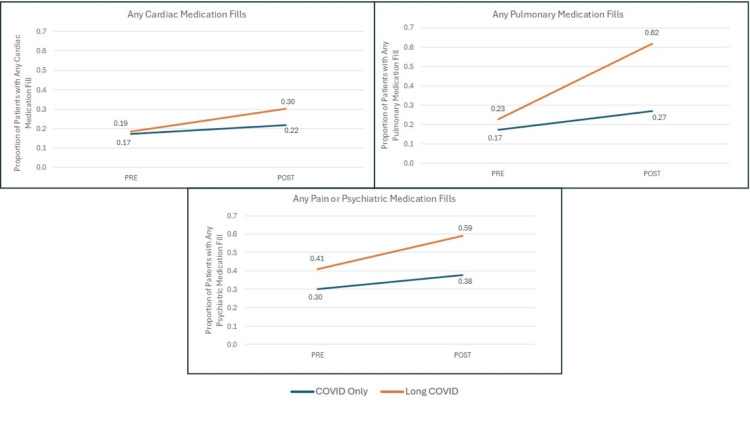
Pre- and Post-Comparisons Between the COVID-Only (NO Long COVID) and Long COVID Groups by Specialty Meds. We compared outcomes during 2019 and the year following COVID infection+28 days (Long COVID window) between those with Long COVID and those with COVID only using inverse probability of treatment weighting (IPTW) propensity score weights in models additionally adjusted for study month, time period, and exposure-time interaction terms to generate difference-in-differences model estimates. P-value on interaction terms in these models was <0.001.

Figure 6 demonstrates the same pattern in the proportions of patients filling at least one prescription for cardiac, pulmonary, and psychiatric medications during each study period.

Complete adjusted results are included in Table 4. The impact of Long COVID diagnosis extended beyond healthcare utilization and into workforce availability ramifications. The adjusted mean number of discrete patient-requested physician release-from-work notes increased more than twofold for Long COVID episodes from pre-COVID to post-COVID while not showing any significant increase in the NO Long COVID group (2.3 [95% CI: 2.2-2.5] post/0.95 [95% CI: 0.86-1.03] pre versus 0.68 [95% CI: 0.67-0.68] post/0.55 [95% CI: 0.54-0.55] pre).

**Table 4:** Adjusted Utilization Comparisons *Number of active medications at encounter nearest the end of the 2019 baseline period or the end of the Long-COVID window DID = difference-in-differences

Utilization	Base Period		Post Period	
DID Model Estimates with 95% CIs	No Long COVID	Long COVID	No Long COVID	Long COVID
All Encounters	12.46 (12.40 – 12.51)	14.10 (13.46 – 14.74)	17.72 (17.66 – 17.79)	29.85 (28.80 – 30.91)
Active Medications*	11.45 (11.41 – 11.49)	13.88 (13.19 – 14.56)	15.59 (15.54 – 15.64)	21.74 (20.92 – 22.56)
Labs Performed	7.74 (7.69 – 7.78)	8.25 (7.79 – 8.71)	9.64 (9.59 – 9.69)	15.60 (14.85 – 16.35)
Imaging Performed	0.50 (0.49 – 0.50)	0.60 (0.56 – 0.63)	0.60 (0.60 – 0.60)	1.04 (0.98 – 1.09)
Video Visits	1.50 (1.49 – 1.51)	1.87 (1.76 – 1.97)	3.32 (3.30 – 3.33)	7.25 (6.92 – 7.57)
Ambulatory Visits	6.05 (6.02 – 6.09)	7.22 (6.78 – 7.65)	5.96 (5.92 – 5.99)	9.24 (8.80 – 9.67)
Secure Messaging Threads	0.44 (0.44 – 0.44)	0.60 (0.56 – 0.65)	0.75 (0.74 – 0.75)	1.47 (1.39 – 1.56)
ED Encounters	0.32 (0.31 – 0.32)	0.38 (0.35 – 0.41)	0.38 (0.38 – 0.38)	0.65 (0.59 – 0.71)
Cardiac Procedures	0.14 (0.14 – 0.14)	0.16 (0.15 – 0.18)	0.15 (0.15 – 0.15)	0.48 (0.45 – 0.51)
Pulmonary Procedures	0.01 (0.01 – 0.01)	0.02 (0.01 – 0.02)	0.01 (0.01 – 0.01)	0.12 (0.11 – 0.13)
Neurological Procedures	0.01 (0.01 – 0.01)	0.02 (0.02 – 0.03)	0.01 (0.01 – 0.01)	0.04 (0.03 – 0.05)
Gastrointestinal Procedures	0.05 (0.05 – 0.05)	0.06 (0.05 – 0.07)	0.05 (0.05 – 0.05)	0.09 (0.08 – 0.10)
Psychiatric Encounters	1.00 (0.98 – 1.01)	1.56 (1.28 - 1.85)	1.42 (1.40 – 1.44)	3.09 (2.73 – 3.44)
Pulmonary Specialist Visits	0.05 (0.05 – 0.05)	0.07 (0.05 – 0.09)	0.06 (0.06 – 0.07)	0.62 (0.55 – 0.69)
Cardiac Specialist Visits	0.16 (0.16 – 0.17)	0.13 (0.10 – 0.16)	0.23 (0.23 – 0.24)	0.43 (0.37 – 0.49)
Neurological Specialist Visits	0.05 (0.05 – 0.05)	0.09 (0.07 – 0.11)	0.07 (0.07 – 0.07)	0.22 (0.19 – 0.26)
Any Cardiac Medication Fill	0.17 (0.17 – 0.17)	0.19 (0.17 – 0.20)	0.22 (0.22 – 0.22)	0.30 (0.29 – 0.32)
Any Pulmonary Medication Fill	0.17 (0.17 – 0.17)	0.23 (0.21 – 0.24)	0.27 (0.27 – 0.27)	0.62 (0.60 – 0.64)
Any Pain or Psychiatric Medication Fill	0.30 (0.30 – 0.30)	0.41 (0.39 – 0.43)	0.38 (0.38 – 0.38)	0.59 (0.57 – 0.61)
Work Activity Status Forms	0.55 (0.54 – 0.55)	0.95 (0.86 – 1.03)	0.68 (0.67 – 0.68)	2.32 (2.17 – 2.47)

## Discussion

This retrospective study demonstrates the broad impact of Long COVID on an integrated healthcare system. Unadjusted utilization of services across multiple domains was significantly higher following COVID episodes resulting in Long COVID in both the pre and post study periods compared with NO Long COVID controls, and these trends persisted after DID adjustment with propensity weighting. Our results are consistent with observations in other settings [2,6,7].

The healthcare cost ramifications of our findings are significant. As an illustrative example, looking at just a single specialty test (e.g. the TTE (Current Procedural Terminology® (CPT) 93306) that may be performed in Long COVID patients with cardiac symptoms such as orthostatic hypotension), we estimate an increased cost in the Long COVID population of approximately $75,000 based on 2025 Medicare fee schedule [13,14]. Similarly, for pulmonary function testing (Table 5) we estimate a minimum excess cost of $18,000. Consistent with our findings, a 2023 cost analysis estimated that patients with Long COVID could face additional healthcare costs ranging from $9,000 to $15,000 annually due to the increased utilization of services such as physical therapy, medications, and specialist consultations [3].

**Table 5:** Current Procedural Terminology (CPT) Codes Used Data derived from the CPT® 2024 Professional Edition [15].

CPT	Procedure
93306	Complete transthoracic echocardiogram (TTE)
94010	Spirometry test
94060	Spirometry with bronchodilator
94200	Maximum voluntary ventilation (MVV) testing
94375	Respiratory flow volume loop
94726	Plethysmography test

Our study also demonstrates the collateral impact of Long COVID and its impact on functionality in the workplace. The incidence of release from work forms for Long COVID episodes was twofold higher than the NO Long COVID episodes, and this likely underrepresents the burden that Long COVID has had on productivity outside of the healthcare domain.

Our results support the need for the development and implementation of outpatient clinics and programs that focus specifically on the unique clinical needs of patients with Long COVID. These clinics typically involve collaboration between internal medicine physicians, various specialists, physical and occupational therapists, mental health professionals, and social workers. Patients often require ongoing support and follow-up care, as symptoms can fluctuate, and new issues may arise over time. Such multidisciplinary resources have been deployed in our system and have shown promise in others [16,17]. Support classes in our system are staffed by primary care clinicians with Long COVID expertise and facilitated by a clinical psychologist and cover multiple Long COVID symptoms and domains including brain fog and postural hypotension.

Access to such services may not be consistent across systems and patient types and as such, there is concern that disparities in the care of Long COVID patients may exist [18].

Limitations

Our study has several limitations. First, it was conducted in a discrete health system within a specific geographic region and therefore may not be generalizable to other locales or systems. Nonetheless, our results are consistent with findings from other systems [2,6,7]. Similarly, our analysis compared utilization differences through 2023, and temporal trends may have evolved since then as they were observed to do so with COVID-19 related utilization between 2019-2021 [19]. The definitions we used in this study have inherent limitations. Our Long COVID cohort was defined by criteria associated with a diagnosis of Long COVID by a clinician and as such our study has a potential care-seeking bias. Episodes with occult Long COVID were not captured in this comparison cohort but may have been included as NO Long COVID episodes. We used adjustment with propensity scoring with inverse probability weighting to address this potential confounder but cannot be certain about the causality of the increased utilization we observed with Long COVID care-seeking patients. It is possible that baseline patient characteristics, such as anxiety or some other driver of more broad care-seeking behavior, may have distorted the parallel trends assumption and contributed meaningfully to our findings. We also could not include the clinical severity of the index COVID episode in our propensity models as it was not available in the baseline (pre-COVID) time period. As with all observational studies, we cannot rule out unmeasured confounding. Similarly, we used a symptom duration definition for our Long COVID group of 28 days that was in use within our system at the time of our study but is shorter than that used or recommended by some guidelines and evolving consensus [20,21]. In a secondary analysis of patients enrolled in a prospective international COVID-19 registry, symptom duration criteria significantly impacted Long COVID prevalence rates [22]. In our study, patients who had extended COVID symptoms greater than 28 days but less than 12 weeks were included in the Long COVID comparison group, and their inclusion likely diluted the magnitude of our health utilization outcomes because this subset of patients had a shorter overall duration of care-seeking for Long COVID type symptoms. Finally, we were not powered to study the impact of Long COVID clinics and support classes on the utilization patterns of Long COVID patients.

## Conclusions

In an integrated delivery system in California, patients whose COVID episode resulted in Long COVID were more likely to have higher healthcare utilization both prior to and after their Long COVID diagnosis compared with patients with a COVID episode and NO Long COVID. Healthcare utilization increased more sharply following Long COVID episodes across nearly all outcomes.
